# Effects of phosphorus enrichment on *Daphnia*–algae interactions in laboratory microcosms

**DOI:** 10.1093/plankt/fbaf002

**Published:** 2025-03-01

**Authors:** Paul C Frost, Kyle DaSilva, Joseph A Frost-Xenopoulos, Wesley S Burr, Catriona L C Jones

**Affiliations:** Department of Biology, Trent University, Peterborough, Ontario K9L 0G2, Canada; Department of Biology, Trent University, Peterborough, Ontario K9L 0G2, Canada; Department of Mathematics & Statistics, Trent University, Peterborough, Ontario K9L 0G2, Canada; Department of Mathematics & Statistics, Trent University, Peterborough, Ontario K9L 0G2, Canada; Environmental and Life Sciences Graduate Program, Trent University, Peterborough, Ontario K9L 0G2, Canada

**Keywords:** ecological stoichiometry, herbivore, nutrient limitation, nutrient recycling, model ecosystems

## Abstract

We examined the effects of phosphorus (P) on algal biomass and stoichiometry and, subsequently, alterations to zooplankton populations. We modified P supply in microcosms and tracked changes in algal and *Daphnia* populations, and phosphorus concentrations. Even though we found algal biomass increased over the experiment in low P containers, greater food abundance did not increase *Daphnia* abundance. In low P containers, a high algal biomass was accompanied with elevated C:P ratios, very low soluble reactive P concentrations and low *Daphnia* fecundity. High algal C:P ratios and low soluble reactive P concentrations in these microcosms indicated a strong P-limitation of algae and food quality constraints on consumer populations. In high P containers, algal biomass initially increased, which led to an increased *Daphnia* abundance. In most of the high P microcosms, rapid increases in *Daphnia* populations led to reduced algal biomass and increased concentrations of soluble reactive P. However, in an outlier high P container, we found a different pattern with elevated algal biomass, low soluble reactive P concentrations and a very large *Daphnia* population. Our results show that P supplies can strongly affect *Daphnia*–algae interactions, but the nature of these effects are likely complicated by internal feedbacks that affect the gain and loss of both populations.

## INTRODUCTION

Grazer–producer interactions can be strongly shaped by the supply of nutrients in the environment (Andersen *et al.*, 2007; Hall *et al.*, 2007). One way that nutrient deprivation constrains the performance of grazers and their population sizes is through the generation of consumer-food elemental imbalances (Sterner and Elser, 2002). Stoichiometrically explicit predator–prey models have been developed and incorporate elemental imbalances by making grazer performance and population growth a function of both food quantity and quality (e.g. Loladze *et al.*, 2000). Such models predict complicated and complex dynamics of grazers across gradients of nutrient enrichment due to interactive effects between resource supply ratios, algal producer stoichiometry, and grazer growth and reproduction (Elser *et al.*, 2012). Mechanisms underlying these dynamics have been seen in small and large-scale experiments where low P supply produces P-limited algae, which places strong elemental constraints on grazer growth rates (Urabe and Sterner, 1996; Elser *et al.*, 2002; Urabe *et al.*, 2002a). Here we examine consumer–producer dynamics using an experimental system (*Daphnia*–algae–phosphorus) that includes temporal dynamics in producer and grazer population-level responses to nutrient enrichment.

The stoichiometry of grazer–producer interactions is partly controlled by the elemental flexibility of producers. Primary producers, including algae, are widely assumed to be non-homeostatic in their phosphorus (P) content such that changes in the supply of this element leads to changes in cellular P content (Sterner and Elser, 2002; Frost *et al.*, 2005a). This variable P content in algae is captured by their carbon to phosphorus (C:P) ratios with low C:P ratios typically showing P-sufficiency and elevated C:P ratios indicating strong P-limitation (Healey, 1978; Healey and Hendzel, 1979). Nutrient enrichment can increase algal biomass due to its positive effects on the producer’s growth rate, but the magnitude of increased biomass ultimately depends on the balance of gains relative to losses due to grazing and/or sedimentation (Urabe *et al.*, 2002a). Consequently, responses of algal biomass and C:P ratios to P enrichment should reflect both the amount of P supplied and the population size of their herbivore consumers (Andersen, 1997).

The effects of nutrient enrichment on phytoplankton producers are also partly controlled by grazer populations. Changes in zooplankton grazer populations are ultimately a function of animal demographic responses to food quantity and quality (Vanni and Lampert, 1992; Makino *et al.*, 2002). Generally, increased quantities of high-quality food yield faster animal growth and more reproduction from individual consumers (McCauley *et al.*, 1999). High P supply should thus result in larger grazer populations, greater total grazing rates and more nutrients recycled back to dissolved pools (Andersen, 1997). Low P supply, on the other hand, should limit algal population growth and increase C:P ratios, both of which are connected to reduced grazer performance and limits on their population growth (Elser *et al.*, 2002; Makino *et al.*, 2002). Elemental constraints on grazer populations should reduce rates of herbivory and slow nutrient recycling. The attenuation of internal grazer-mediated nutrient feedbacks can result in more algal biomass but of greatly reduced nutrient content (Sommer, 1992; Urabe *et al.*, 2002a).

Trophic interactions between grazers and producers may also vary with the ambient and/or initial conditions of the experiment. For example, reduced supply of light or other growth-limiting elements, at a fixed P supply, can reduce algal C:P ratios and lessen stoichiometric imbalances with consumers (Urabe and Sterner, 1996; Urabe *et al.*, 2002b). The relative biomass of producers and consumers at the start of an experiment could also modify subsequent predator–prey dynamics by affecting how quickly grazer populations exert significant grazing losses on algal populations and, in turn, become significant P recyclers (Andersen, 1997; Loladze *et al.*, 2000). The effects of initial conditions on stoichiometric interactions of grazer–producer interactions could be especially important when compounded over meaningful time scales.

In this study, we examined how P supply affects the stoichiometry of grazer-algal interactions with a model ecosystem. To do so, we manipulated P supply rates (high and low) and tracked changes in algal food resources, grazer populations and the concentrations of inorganic P in microcosms. Our primary objective was to determine how P supply modifies grazer population dynamics by way of changes in algal growth and stoichiometry. This study also provides a demonstration of the usefulness of this model system for future experiments examining the stoichiometry of grazer–producer interactions.

## METHODS

### Starting conditions

Our experimental unit was a microcosm (i.e. rectangular glass tank) filled with five liters of modified COMBO media (Frost *et al.*, 2024). At the start of the experiment, we added COMBO media to replicated tanks (*n* = 4 for each P level) with either a high P (309 μg P/L) or low P (38.6 μg P/L) concentration. After this, we added an algal population (*Scenedesmus obliquus*) to each tank at an estimated starting concentration of ~ 3 mg C/L. Prior to the start of the experiment, algal populations were grown under P-sufficient conditions in culture jars that were diluted 50% each day to maintain relatively high growth rates of algal cells. On the first day of the experiment, we collected algae from the culture jars and used a centrifuge to remove COMBO media and concentrate cells. Algal pellets were resuspended in N- and P-free COMBO at a higher density. Small subsamples of this concentrated algal slurry were dried for 2 hrs at 60°C and weighed to estimate the dry mass concentration. We used this dry mass estimate to determine the volume of algal slurry to add to tanks to produce the desired starting biomass. After filters were dried for an additional 24 hrs at 60°C, we placed them in the freezer at −20°C until subsequent measurement of their algal C content (see Methods below). Based on subsequent algal C measurements and the volume of slurry that we added, we estimated that the starting algal biomass for this experiment was 2.97 mg C/L. After the addition of algae to each tank, we added 20 individual *Daphnia pulex* that had been rinsed with N- and P-free COMBO. Founder *Daphnia* were 5 days old at the start of the experiment and all had been borne to clonal sisters maintained under conditions of high food abundance.

### Maintenance and sampling of microcosms

Over the course of the experiment, we sampled experimental tanks every 2 days by removing 10% of the tank volume. We replaced this volume and returned the tank volume to 5 L with modified COMBO having a high P (309 μg P/L) or low P (51.5 μg P/L) concentration. To sample, we first gently mixed the tank’s water and resuspended settled algae by stirring the entire tank several times. We then removed 500 mL of water by dipping a large beaker into the center of the experimental tank. Sampled water was immediately poured through an 80-μm mesh to remove *Daphnia*. Mesh-filtered water was then processed for chlorophyll (CHL), particulate CN and P, and soluble reactive P (SRP). For chlorophyll and particulate CN and P samples, we filtered 50 mL of collected water onto pre-ashed glass fiber filters (GF/F). GF/F filters were then immediately frozen for CHL or dried for 48 hr at 60°C for CN and P. After drying, CN and P filters were placed into the freezer until further analysis. Two subsamples of filtered water that had passed through the GF/F were saved frozen until analysis for SRP. Samples for CHL and particulate CN and P were saved every fourth day. SRP samples were saved every second day.

### Daphnia preservation and counting

After rinsing animals off the 80-μm mesh filter, we placed them in 100% ethanol for 30 seconds. We added deionized water to dilute the ethanol to 70% and stored animals in this diluted ethanol at room temperature (Black and Dodson, 2003). To estimate population size, we counted all *Daphnia* in each sample using a dissecting microscope at 40× magnification. In addition, we recorded the presence and number of eggs in each of the first 20 individual *Daphnia* encountered in each sample. *Daphnia* samples were saved every second day during each dilution event.

### Chemical analysis

To estimate CHL, we placed frozen filters into 20-mL ethanol in the dark for 24 hrs at 4°C. After this period, extracted chlorophyll was measured on a Turner spectrofluorometer calibrated with known standards (Marker, 1994). We measured algal C and N on another set of GFF filters using a Vario Cube CN analyser (Elementar Americas). Algal P was measured on the last set of filters following digestion in the autoclave with 5% potassium persulfate (APHA, 1992). We then analysed P concentrations of digested samples using the molybdate blue reaction (APHA, 1992). SRP was also analysed on filtered water with ascorbic acid-molybdate blue reaction.

### Statistical analysis

We assessed the effects of P-supply on algae and *Daphnia* dynamics by fitting second-order polynomial regression models to the experimental results. As the observations for each individual tank were not independent through time, this was considered a repeated measures design with tank-level error structures included in fitted models. These models also included interaction terms between treatment and time to evaluate if P-supply differentially altered responses over time. One high P tank was identified as an outlier and was excluded from all analyses, as this replicate was found to be well outside the standard deviation of the other three tanks within this treatment and exhibited highly asynchronous dynamics. Data were natural log transformed prior to analysis when appropriate. In addition, we assessed statistical differences in *Daphnia* population growth and fecundity metrics by determining the overlap of 95% confidence intervals of these response variables between high and low P supply tanks. For this analysis, we calculated population growth rate as follows:


$$ population\ growth\ rate=\frac{\left(\ln final\ density\right)-\left(\ln initial\ density\right)}{number\ of\ days} $$

using the density data on Days 0 and 20. In addition, we calculated the average population size using data only for the last 10 days of the experiment.

## RESULTS

### Algal biomass

**Fig. 1:**
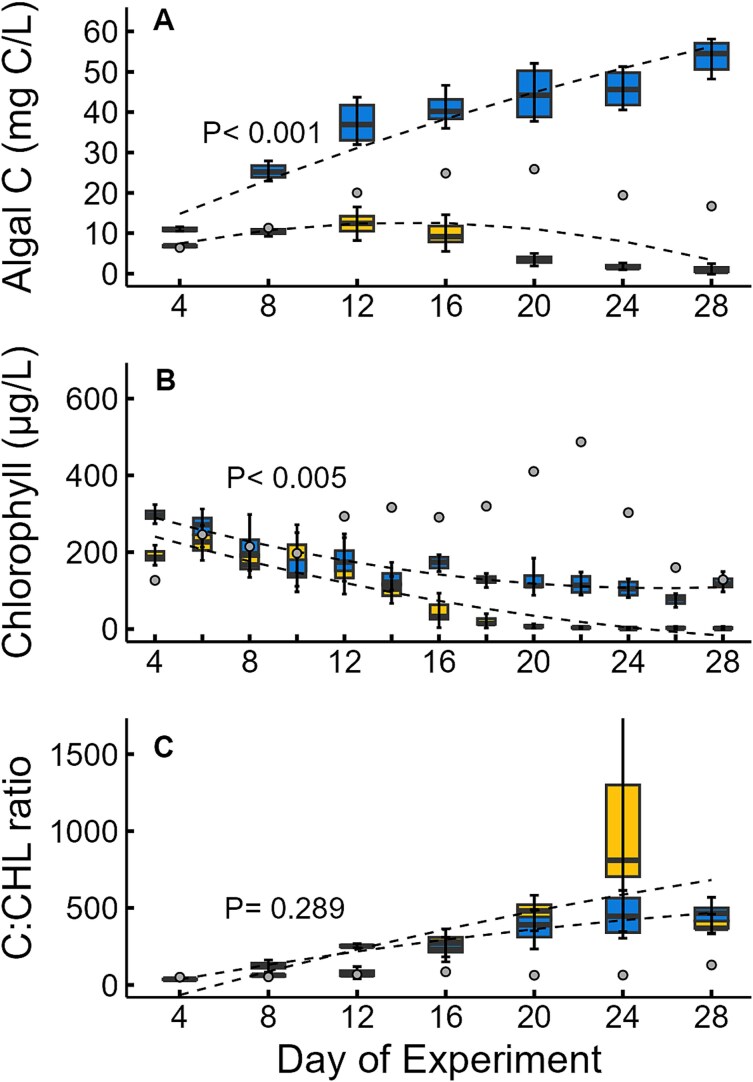
Algal carbon (C) concentrations (**A**), chlorophyll (Chl) concentrations (**B**) and C:Chl ratios (**C**) in tanks receiving high P and low P supply rates over the duration of the experiment. Treatments are denoted by blue (low P supply), yellow (high P supply) and outlier tank (gray dot). Shown are the means (solid line in boxes), 25–75 quantiles (boxes) and 95% confidence intervals (error bars) among replicate tanks on each sampling event (excluding the outlier tank for the high P supply bars). *P*-value indicates the level of significance between fitted polynomial lines (dotted lines). See Supplementary Table 1 for details of these regression fits.

There was a significant interactive effect of time and P-level on algal C concentrations (Fig. 1). While algal C concentrations increased in all tanks over the first third of the experiment, this increase was different between the two P-levels. In low P tanks, algal C reached a concentration of ~ 40 mg C/L after 8 days, which was about 4-fold higher than seen in the high P tanks (Fig. 1). Algal C in the low P tanks continued to increase over the course of experiment and eventually reached concentrations approaching ~ 50 mg C/L (Fig. 1). During the last 20 days of the experiment, algal C decreased in three of the high P tanks to very low concentrations (Fig. 1). In the other high P tank, algal C increased over the first 20 days to about 25 mg C/L and then marginally decreased over the last week of the experiment (Fig. 1).

There were also interactive effects of time and P-level on CHL (Fig. 1). Under low P supply, CHL declined over the experiment, but concentrations remained at or above 50 ug/L for the last half of the experiment (Fig. 1). CHL in three of the four high P tanks were relatively high (~150–200 μg/L) for the first week and then decreased to nearly undetectable levels by Day 20 (Fig. 1). The other high P tank, which was identified as an outlier due to divergent algal C results, also exhibited a much different CHL time sequence from the other three replicate high P tanks. In this divergent tank, CHL increased for much of the experiment and reached a maximum concentration of ~ 500 μg/L on the 24th day of the experiment (Fig. 1). There was no significant interaction term for C:CHL ratios, which generally began low (50 or below) and increased over the experiment with maximum values in the 300–600 range in nearly all of the tanks (Fig. 1). The divergent high P tank produced C:CHL ratios at or below 100 for most of the experiment (Fig. 1).

### Algal C:N:P ratios

**Fig. 2:**
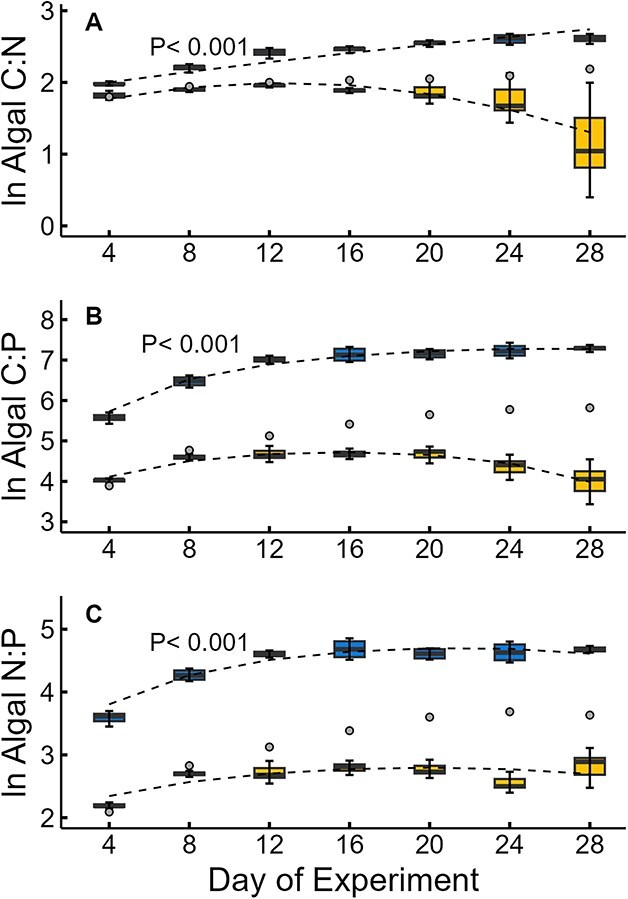
Natural log transformed algal C:N ratios (**A**), C:P ratios (**B**) and N:P ratios (**C**) in tanks receiving high P and low P supply rates over the duration of the experiment. Treatments are denoted by blue (low P supply), yellow (high P supply) and outlier tank (gray dot). Shown are the means (solid line in boxes), 25–75 quantiles (boxes) and 95% confidence intervals (error bars) among replicate tanks on each sampling event (excluding the outlier tank for the high P supply bars). *P*-value indicates the level of significance between fitted polynomial lines (dotted lines). All ratios presented by moles. See Supplementary Table 1 for details of these regression fits.

For all three ratios (C:N:, C:P and N:P), there were significant interactive effects of time and P-level (Fig. 1). In three of the high P tanks, algal C:N ratios remained below 8 for most of the experiment, with two tanks decreasing to <4 on after Day 24 (Fig. 2). The outlier high P tank showed a moderate increase in C:N ratios over the last 10 days of the experiment (Fig. 2). Low P supply produced much higher algal C:N ratios over the experiment with values eventually reaching ~ 12–14 during the last week of the experiment (Fig. 2). In three of the high P tanks, algal C:P ratios were at or below the Redfield ratio (~100) over the entire experiment (Fig. 2). The outlier high P tank once again exhibited a different pattern with C:P ratios increasing to values above 300 during the last week of the experiment (Fig. 2). Low P tanks, on the other hand, exhibited increased C:P ratios in the algae with values exceeding 1000 by the 12th day (Fig. 2). These high algal C:P ratios were maintained in low P tanks over the remainder of the experiment with values reaching the 1200–1400 (Fig. 2). Algal N:P ratios largely tracked patterns seen for C:P ratios with high P tanks showing lower values (<20) and low P tanks having much higher N:P ratios (>100) for the last half of the experiment (Fig. 2). The outlier high P tank again showed a different pattern with N:P ratios increasing to close to 40 by the end of the experiment (Fig. 2).

**Fig. 3:**
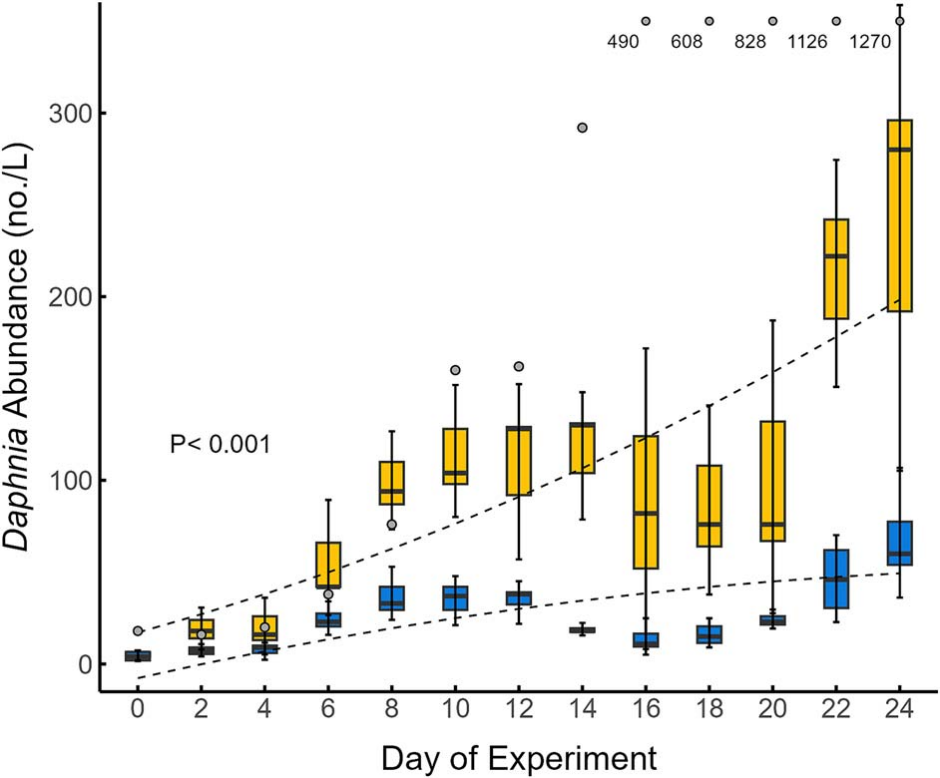
*Daphnia* abundance in tanks receiving high P and low P supply rates over the duration of the experiment. Treatments are denoted by blue (low P supply), yellow (high P supply) and outlier tank (gray dot). Shown are the means (solid line in boxes), 25–75 quantiles (boxes) and 95% confidence intervals (error bars) among replicate tanks on each sampling event (excluding the outlier tank for the high P supply bars). *P*-value indicates the level of significance between fitted polynomial lines (dotted lines). See Supplementary Table 1 for details of these regression fits.

### Daphnia abundance and fecundity

We also found that changes in *Daphnia* abundance through time varied with P-supply (Fig. 3). In all tanks, the abundance of *Daphnia* was relatively low (four animals/L) on the day that the experiment started. While *Daphnia* population abundance increased in all tanks over the 12 days that followed (Fig. 3), these increases were greater in high P tanks, which on Day 12 contained about 100 animals/L. *Daphnia* abundance in three of the high P tanks stayed in this range until the last two sampling dates when abundance increased over 200 animals/L. The divergent high P tank with higher algal biomass exhibited much greater and continuous increases in *Daphnia* abundance over the entire experiment with a peak abundance of > 1200 *Daphnia*/L. We found low P tanks had smaller *Daphnia* populations, which remained below 100 animals/L for the entire experiment (Fig. 3).

**Fig. 4:**
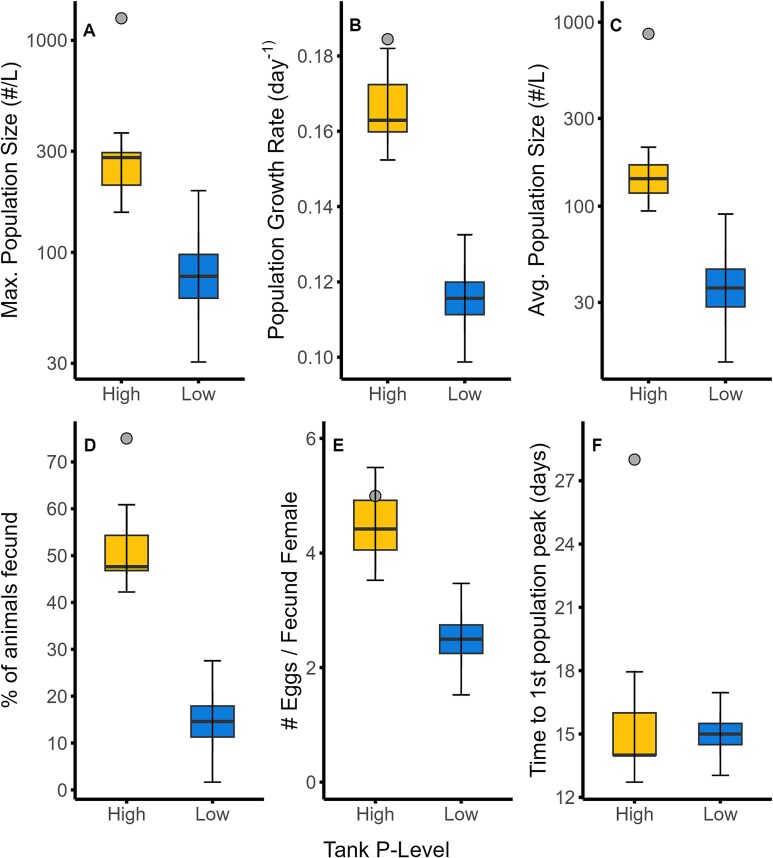
Maximum population size (**A**), population growth rate (**B**), population size (averaged over last 10 days) (**C**), % of animals fecund (averaged over last 10 days) (**D**), number of eggs per *Daphnia* (**E**), and time to first population peak (**F**) in high and low P tanks. Shown are the means (solid line in boxes), 25–75 quantiles (boxes) and 95% confidence intervals (error bars) among replicate tanks on each sampling event (excluding the outlier tank for the high P supply bars; shown with a gray dot).

We found multiple aspects of *Daphnia* populations and demography to vary significantly between the high P and low P tanks. The maximum population size was, on average, about 3 times higher in high P tanks compared to the low P tanks (Fig. 4). In the outlier high P tank, the maximum population size was 1270 animals/L, which was about five times higher than the average of the other three high P tanks. Similarly, the average population size over the last 10 days of the experiment was 4 times higher in high P tanks than low P tanks with the divergent high P tank nearly an order of magnitude higher (Fig. 4). These differences in population size were preceded by rapid growth of all *Daphnia* populations over the first 14 days of the experiment. The population growth rate during this initial period was higher in the high P tanks than the low P tanks (Fig. 4). Despite these differences in population growth rate, the number of days to the first population decline was approximately the same for both high P and low P tanks (Fig. 4), with the exception of the divergent high P tank which had increasing population size until nearly the end of the experiment.

**Fig. 5:**
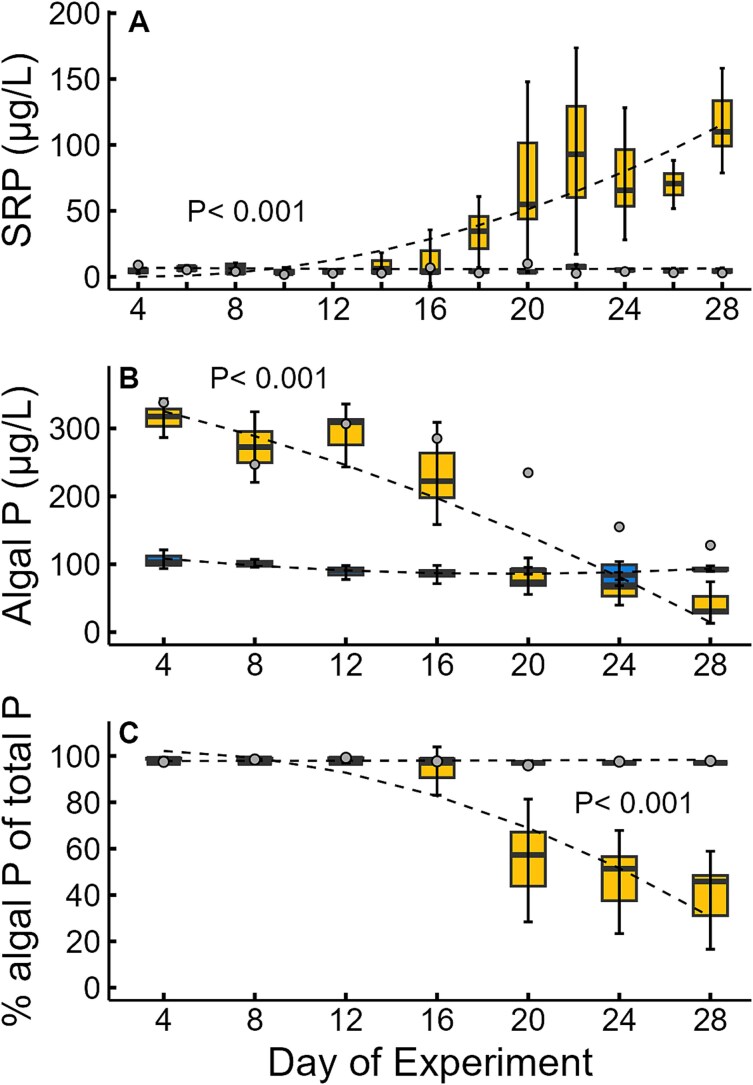
Soluble reactive P concentration (**A**), algal P concentration (**B**) and % of total P in algal pool (**C**) in tanks receiving high P and low P supply rates over the duration of the experiment. Treatments are denoted by blue (low P supply), yellow (high P supply) and outlier tank (gray dot). Shown are the means (solid line in boxes), 25–75 quantiles (boxes) and 95% confidence intervals (error bars) among replicate tanks on each sampling event (excluding the outlier tank for the high P supply bars). *P*-value indicates level of significance between fitted polynomial lines (dotted lines). See Supplementary Table 1 for details of these regression fits.

We also found that fecundity differed between the high P and low P tanks over the last 10 days of the experiment. In high P tanks, about 50% of *Daphnia* were carrying eggs during this time period whereas less than 20% of animals were carrying eggs in the low P tanks (Fig. 4). The divergent high P tank had more than 60% of *Daphnia* being fecund on each sampling date over the last half of the experiment. We also found the number of eggs per fecund female was higher in all four of the high P tanks compared to low P tanks for the last 10 days of the experiment (Fig. 4).

### Phosphorus concentrations

There was also a significant interactive effect between time and P-level on the responses of SRP, algal P and the % algal P of total P (Fig. 5). In three of the four high P tanks, SRP was low up to Day 14 and after that increased up to nearly 100 μg/L (Fig. 5). The outlier high P tank and all of the low P tanks had low SRP concentrations for the entire experiment (Fig. 5). Concentrations of algal P declined in all the high P tanks over the duration of the experiment (Fig. 5). In the low P tanks, algal P concentrations remained low for the entire experiment (Fig. 5). Despite these low algal P concentrations, the % of total P in the algal P pool was quite high (>90%) in low P tanks due to the very low concentrations of SRP. In the high P tanks, the % of total P in the algal pool decreased after Day 20 due to the reduction in algal biomass and the increase in SRP (Fig. 4). The exception was the divergent high P tank, which showed P dynamics similar to the low P tanks with nearly 100% of total P found in the algal P pool for the entire experiment (Fig. 4).

## DISCUSSION

We examined the interactions between inorganic P supply, algae and zooplankton grazers in microcosms receiving high and low P supply rates. In general, higher P led to increased food quality, increased grazer abundance and subsequent reductions in algal biomass. This eventually led to reduced algal demand for P and a build-up of the inorganic P pool. Low P supply quickly produced strongly and perpetually P-limited algae, which resulted in lower *Daphnia* abundance. The high algal biomass and low grazer density produced by low P concentrations were accompanied by low inorganic P concentrations over the experimental duration. Overall, reducing P supply in our experimental containers resulted in higher algae and lower grazer biomass. This dynamic matches results from previous experimental studies (Urabe and Sterner, 1996; Urabe *et al.*, 2002a) and mathematical models (Loladze *et al.*, 2000), where animal performance and population size become decoupled from food abundance at low P supplies. Our results are further demonstration that low inorganic P supply rates can reduce elemental food quality and constrain grazer performance with effects on their population growth.

Algal biomass increased in all tanks, regardless of P supply, during the first few days of our experiment. This increase was faster for low P tanks immediately following the start of the experiment. The slower growth of high P algae may seem inconsistent with the known ability of inorganic nutrients to increase growth of plants and algae. However, very high P concentrations can slow initial algal growth as these cells likely prioritize short-term P uptake and storage over immediate growth (Solovchenko *et al.*, 2019). This lag in algal population growth was likely absent in the low P tanks where inorganic P would have been quickly reduced to very low concentrations that prevented the algae from employing luxury P uptake. With grazer abundance low, algal loss rates were likely minimal during this initial stage of the experiment, which also contributed to increased algal biomass. After an initial growth period, algal biomass declined rapidly in high P tanks coinciding with increasing *Daphnia* abundance and presumably, higher community grazing rates. In contrast, algal biomass continued to increase in low P tanks over the course of the experiment likely due to the low *Daphnia* abundance and limited losses to grazing (Boersma and Meunier, 2020). These changes in algal biomass show phosphorus supply can strongly affect gain and loss processes by directly affecting algal growth and indirectly by controlling the population size of the grazer.

While chlorophyll concentrations are often used to track algal biomass in lakes, we saw a decoupling of algal C and chlorophyll concentrations in our experiments. This disconnect between algal C and chlorophyll was reflected by the increases in C:CHL ratios observed in all tanks. Chlorophyll concentrations generally decreased in all tanks over the entire experiment regardless of changes in algal C. This may have partly resulted from algal cells elevating their C:CHL ratios in response to acute P-limitation in the low P tanks. C:CHL ratios are known to widely vary within algae due to physiological acclimation to changes in light, temperature and nutrients (Geider *et al.*, 1997). The very high C:CHL ratios observed in our experiment (>300) are at or above high values previously reported from measurements of pure algal cultures (e.g. Geider, 1987). Given this, it is likely that the accumulation of non-algal C (e.g. bacterial or non-living detrital C), contributed to the elevated C:CHL ratios (Frost *et al.*, 2005b). This mechanism was also probably responsible for elevated C:CHL ratios in the high P tanks where algal densities were very low for much of the last half of the experiment.

Algal C:N, C:P and N:P ratios also all varied between high and low P supply in our experiment. We found low P supply led to increasing algal C:N, C:P and N:P ratios as is normally observed in P-limited algal cultures and phytoplankton bioassays (Sterner and Elser, 2002; Frost *et al.*, 2023). Algal C:P ratios in low P tanks quickly exceeded 200 and eventually reached values over 1200. These numbers greatly exceed the threshold C:P ratio for *Daphnia* growth (~150; Khattak *et al.*, 2018) and likely placed extreme P-constraints on grazer performance. Lower C:P ratios were found in the high P tanks, which is expected due to the higher P availability, the relatively high P recycling rates and low algal densities produced by intense grazing (Sommer, 1992; Elser and Urabe, 1999). The somewhat higher algal C:P ratio (~300) seen in the divergent high P tank likely reflects sustained moderate P-limitation that resulted from the higher quantities of algal biomass that we observed. These patterns in algal C:N, C:P and N:P ratios, through time and between treatments, reflect combined effects of P-supply (external and internal), algal growth rates and grazing losses, with effect strengths of each factor presumably varying between high and low P supply treatments and across the 4 weeks of this experiment.

We found very different grazer population dynamics between systems receiving high and low P supply. In general, animal population size increases when births exceed deaths, which are both a function, at least partly, of food quantity and quality (Persson *et al.*, 2008; Bi and Sommer, 2020). During the early portion of our experiment, births would have predominated with deaths mostly limited to losses during each sampling event. In high P tanks, better algal food quality led to earlier and greater reproduction as evidenced by the high proportion of fecund animals and greater average number of eggs in fecund animals. Both metrics were lower in *Daphnia* sampled from low P tanks, which would be expected if P-limitation in the low P tanks constrained growth rates of these animal populations. Later in the experiment (after 10–14 days), the size-structure of daphnid populations likely shifted away from older and larger animals to an increased contingent of smaller and younger animals. As size structure was not assessed with in this experiment, it is unknown whether it varied with P supply or over the course of the experiment. Future studies should track the size-structure of experimental populations to better determine how P-supply affects demographic properties of grazer populations over longer time periods.

One additional aspect of the experimental system that we tracked was the distribution of P between dissolved and algal fractions. Most of the P was found in algal biomass in the low P tanks for the entire experiment. This is consistent with severe P-limitation in these algal populations, which would greatly reduce the residence time of phosphate ions (new or recycled) in the water before moving back into the algal pool (Hudson *et al.*, 1999). The high proportion of P located in the algal pool was also seen in tanks receiving high P supply over the first half of the experiment after which dissolved P began to accumulate in the water. This change in P speciation in the tanks was associated with low algal biomass resulting from high grazer biomass. In other words, high P concentrations led to greatly increased grazer biomass that resulted in less algal biomass and less total P uptake. The divergent high P tank again showed different results whereby no increases in dissolved P were observed and nearly all P was found in the algal pool.

Our results show highly synchronous behavior of four low P replicate tanks and three of the four high P tanks. While this indicates that we controlled most of the potentially influential variables in our experiment, the divergent behavior of one replicate tank under high P conditions indicates that some aspect of this tank differed from the other three replicates. Previous work by Urabe *et al.* (2002b) also found asynchronous dynamics with a single replicate potentially due to differences in light intensity. One possible explanation for the outlier replicate in this study has to do with the first sampling of *Daphnia* on Day 4. As we removed the same volume of media from all four tanks, the same death rate should have been imparted on each animal population. However, we inadvertently (and randomly) removed more animals from the outlier tank on the first sampling day. This larger reduction in population size may have limited early population growth and by extension reduced subsequent grazing rates on the algae. This would be consistent with the greater gains in algal biomass after 10 days and the sustained mild P-limitation in this tank. If so, differences in starting conditions, including even relatively subtle differences in *Daphnia* population size, may produce highly variable results in this grazer–producer system. This type of dynamic interaction between grazers and producers are generally predicted by stoichiometric models (Loladze *et al.*, 2000), where differences in grazer abundance affects consumptive losses of algal biomass, alters per capita P supply rates to algae, and can, in turn, affect their own population growth (Sommer, 1992). Future use of this or similar experimental systems should be aware of these feedbacks and the need to carefully control grazer, producer and nutrient conditions, especially during the early phases of the experiments. It is also worth noting that we saw an order of magnitude more *Daphnia* in the population of the divergent high P tank. This elevated population size was likely only possible due to the combination of both high food abundance and good food quality (but not necessarily ideal) that resulted from initially low *Daphnia* biomass. In comparison, it appears that food quantity became severely limiting to *Daphnia* populations in the other three high P tanks, whereas food P content was too low to sustain population growth in the low P supply tanks.

There are many potential experiments that could use this grazer–producer system to explore animal and algal population responses to light, nutrients and temperature. Most previous experimental studies of the stoichiometric controls on zooplankton populations focused on light intensity or light:nutrient ratios (e.g. Sterner *et al.*, 1998; Elser *et al.*, 2002; Urabe *et al.*, 2002a; Urabe *et al.*, 2002b) and used widely varying container size and experimental duration. There are many more independent variables that could be manipulated, which include the concentrations and ratios of essential nutrients (including N, P, Fe, Ca or other trace elements/dissolved ions), light quality, temperature, algal species and diversity, initial algal biomass, animal community composition and diversity, animal death rates and initial animal biomass. Sequential and/or combinational manipulation of these variables would allow for interactive effects among these variables to be assessed on zooplankton grazer populations and would deepen our phenomenological knowledge of animal population dynamics, especially under different nutrient supply rates and ratios. While it would also be valuable to extend experimental duration to permit assessment of multiple cycles of grazer–producer biomass, the length of experiment should be carefully considered to avoid container effects previously documented for small sized microcosms (e.g. Smith *et al.*, 2005).

This work could be coupled to stoichiometric models (e.g. Andersen, 1997; Loladze *et al.*, 2000) that track the amount and distribution of nutrients and biomass between compartments in this two-trophic-level foodweb. Ideally, this experimental system could better inform existing models and serve as a testing ground for novel predictions that emerge from these stoichiometrically explicit mathematical frameworks (e.g. Loladze *et al.*, 2000). As an experimental system, it is obvious that there are aspects that highly unrealistic (e.g. algal biomass reaching 50 mg C/L) and these differences need to be acknowledged, if and when, these results are used to understand ecological patterns in nature.

## CONCLUSIONS

We examined algal and *Daphnia* population responses to two levels of P supply with a month-long experiment. This time frame allowed for internal feedbacks to operate and modify relationships between P concentrations, algal biomass and grazer abundance. Low P resulted in more algae having less P, which limited growth of grazer populations and their ability to reduce algal biomass and increase internal P supplies. Higher P supply resulted in higher algal food quality and more *Daphnia*, which led to large reductions in algal biomass and accumulation of inorganic P in these systems. While the results of more algal biomass with less P may seem counterintuitive, they are consistent with stoichiometric predictions that incorporate the effects of variable P supply rates on grazer–producer interactions. Future use of this experimental system could include more complicated designs, more treatment types and track more variables through time to better understand how stoichiometric mechanisms affect interactions between grazers and their algal food.

## Supplementary Material

Supplementary_Table_1_fbaf002

## Data Availability

The data used in this paper are available at: https://doi.org/10.6084/m9.figshare.26260349.v1
