# Supplementary material for: Effects of phosphorus enrichment on Daphnia–algae interactions in laboratory microcosms
Source: J Plankton Res. 2025 Mar 1;47(2):fbaf002. doi: 10.1093/plankt/fbaf002 (PMC11879204; doi:10.1093/plankt/fbaf002)
Supplement: Supplementary_Table_1_fbaf002 [file supplementary_table_1_fbaf002.docx]

**Supplementary Table 1**. Statistics for quadratic regressions (Y = Intercept + b_1_ X + b X^2^) fit to measured response variables.

| **Response** |  | **Intercept** | **b_1_** | **b_2_** |  |  |  |
| --- | --- | --- | --- | --- | --- | --- | --- |
| Algal C | High P | 2.57 | 1.41 | -0.05 |  |  |  |
|  | Low P | 5.7 | 2.35 | -0.02 |  |  |  |
|  |  |  |  |  |  |  |  |
| Chlorophyll | High P | 275.9 | -9.10 | 0.06 |  |  |  |
|  | Low P | 325.6 | -9.34 | 0.09 |  |  |  |
|  |  |  |  |  |  |  |  |
| C:CHL | High P | -232 | 43 | -0.37 |  |  |  |
|  | Low P | -69.2 | 27.5 | -0.30 |  |  |  |
|  |  |  |  |  |  |  |  |
| Ln algal C:N | High P | 1.52 | 0.075 | -0.003 |  |  |  |
|  | Low P | 6.5 | 0. 26 | -0.001 |  |  |  |
|  |  |  |  |  |  |  |  |
| Ln algal C:P | High P | 12.7 | 11.80 | -0.38 |  |  |  |
|  | Low P | 3.2 | 0.67 | -0.03 |  |  |  |
|  |  |  |  |  |  |  |  |
| Ln algal N:P | High P | 1.66 | 0.57 | -0.43 |  |  |  |
|  | Low P | 1.95 | 1.56 | -0.53 |  |  |  |
|  |  |  |  |  |  |  |  |
| *Daphnia* abundance | High P | 7.95 | 2.14 | 0.029 |  |  |  |
|  | Low P | -15.2 | 2.08 | -0.015 |  |  |  |
|  |  |  |  |  |  |  |  |
| SRP | High P | 0.91 | -0.43 | 0.05 |  |  |  |
|  | Low P | 7.15 | -0.09 | 0.001 |  |  |  |
|  |  |  |  |  |  |  |  |
| Algal P | High P | 355 | -6.85 | -0.19 |  |  |  |
|  | Low P | 122 | -3.74 | 0.09 |  |  |  |
|  |  |  |  |  |  |  |  |
| %PP | High P | 101.45 | 0.64 | -0.11 |  |  |  |
|  | Low P | 98.02 | 0.001 | 0.0001 |  |  |  |
|  |  |  |  |  |  |  |  |
